# Evaluation of Protective Efficacy of Respiratory Syncytial Virus Vaccine against A and B Subgroup Human Isolates in Korea

**DOI:** 10.1371/journal.pone.0023797

**Published:** 2011-09-07

**Authors:** Ji-Eun Jang, Jee-Boong Lee, Kyung-Hyo Kim, Sung Moo Park, Byoung-Shik Shim, In Soo Cheon, Man Ki Song, Jun Chang

**Affiliations:** 1 Division of Life and Pharmaceutical Sciences, Center for Cell Signaling and Drug Discovery Research, Ewha Womans University, Seoul, Korea; 2 Department of Pediatrics, Center for Vaccine Evaluation and Study, Ewha Womans University School of Medicine, Seoul, Korea; 3 Laboratory Science Division, International Vaccine Institute, Seoul, Korea; University of Georgia, United States of America

## Abstract

Human respiratory syncytial virus (HRSV) is a significant cause of upper and lower respiratory tract illness mainly in infants and young children worldwide. HRSV is divided into two subgroups, HRSV-A and HRSV-B, based on sequence variation within the G gene. Despite its importance as a respiratory pathogen, there is currently no safe and effective vaccine for HRSV. In this study, we have detected and identified the HRSV by RT-PCR from nasopharyngeal aspirates of Korean pediatric patients. Interestingly, all HRSV-B isolates exhibited unique deletion of 6 nucleotides and duplication of 60 nucleotides in the G gene. We successfully amplified two isolates (‘KR/A/09-8’ belonging to HRSV-A and ‘KR/B/10-12’ to HRSV-B) on large-scale, and evaluated the cross-protective efficacy of our recombinant adenovirus-based HRSV vaccine candidate, rAd/3xG, by challenging the immunized mice with these isolates. The single intranasal immunization with rAd/3xG protected the mice completely from KR/A/09-8 infection and partially from KR/B/10-12 infection. Our study contributes to the understanding of the genetic characteristics and distribution of subgroups in the seasonal HRSV epidemics in Korea and, for the first time, to the evaluation of the cross-protective efficacy of RSV vaccine against HRSV-A and -B field-isolates.

## Introduction

Human respiratory syncytial virus (HRSV) is a significant cause of respiratory illness in infants and young children worldwide. HRSV is also a recognized pathogen associated with respiratory tract disease in the elderly and immunocompromised individuals [1], [2]. HRSV causes a mild respiratory infection leading to clinical symptoms such as cough and fever in healthy adults, but serious pulmonary infection including pneumonia and bronchiolitis may occur in young children, elderly and immunodeficient patients [3], [4], [5], [6]. Furthermore, there is some evidence that RSV-induced severe respiratory tract disease in early childhood is associated with the development of asthma later in life [7], [8], [9], [10].

It has been reported that almost all children have been infected with HRSV by the second birthday and approximately half experience re-infections [11], [12]. Repeated infections might occur due to ineffective immunity induced by natural infection and/or partial protection by maternal antibodies [11], [13]. There is currently no licensed HRSV vaccine.

HRSV has been divided into two antigenic subgroups, HRSV-A and HRSV-B, based on reactive patterns to monoclonal antibodies. The most genetic and antigenic variability between subgroups has been found in the G glycoprotein. The G glycoprotein sequence has only 53% homology between subgroups and even exhibits limited diversity within the same subgroup: ∼20% differences in the HRSV-A subgroup and ∼9% in HRSV-B.

In this study, we tested a collection of nasopharyngeal samples from pediatric patients admitted to Ewha Womans University Mokdong Hospital in Korea with acute lower respiratory tract infection (LRTI) between November 2008 and April 2010. In order to detect HRSV, RT-PCR assays were performed with RSV-specific primer sets. The G gene sequences of RSV-positive samples were then analyzed for genetic variability among Korean isolates by construction of a phylogenetic tree. Two of the HRSV isolates, named ‘KR/A/09-8’ (subgroup A) and ‘KR/B/10-12’ (subgroup B), were adapted to HEp-2 cells for large-scale culture. Then, we tested the efficacy of our previously reported RSV vaccine [14], rAd/3xG, against these field-isolated HRSV. The result showed that a single intranasal immunization of rAd/3xG protected the mice completely from KR/A/09-8 challenge and partially from KR/B/10-12 infection, confirming that rAd/3xG could be further developed as a promising vaccine candidate against HRSV infection.

## Methods

### Clinical specimens

Seventy nasopharyngeal aspirates were collected from children under 3 years of age, who were admitted to the Ewha Womans University Mokdong Hospital (Korea) with clinical signs of acute lower LRTI between November 2008 and April 2010. Nasal wash samples were obtained using a sterile catheter. Collected samples were adjusted with additional medium to make total volume of 10 ml, and were filtered through a 0.45-µm filter. Then, samples were used to inoculate HEp-2 cells in 60 mm cell culture dishes at 50∼60% confluency and inoculated cells were observed daily for signs of infection for up to 14 days. Infected cells were harvested, sonicated for 15 min, and centrifuged at 300 g for 10 min and then supernatants were collected and stored at −70°C until further use.

### RNA Extraction, quantitative real-time PCR and RT-PCR assay

RNA was extracted from the infected cell lysates with the RNeasy Mini Kit (Qiagen) according to the manufacturer's instruction, and cDNAs were synthesized from eluted RNA using the ImProm-II Reverse Transcription System (Promega) and RSV-specific primers. The NS2, SH and G genes were chosen for detection of RSV through RT-PCR with specific primers. The following primers are used: NS2-5′ ATTGGCATTAAGCCTACAAAGCA, NS2-3′ CTTGACTTTGCTAAGAGCCATCT; SH-5′ AATTGGAAGCACACAGCTAC, SH-3′ TTGCATTTGCCCCAATGTT; G-5′ ATGATTGCAATACTAAA, G-3′ ACACTGGTATACCAACC. RT-PCR was performed with 1 µg of cDNA in a 20-µl reaction volume. Cycling conditions were 94°C for 3 min, followed by 40 cycles of 94°C for 30 sec, 50°C for 1 min and 72°C for 1 min. Amplification products were analyzed by 1% agarose gel electrophoresis.

### Nucleotide sequencing and phylogenetic analysis

The amplified products of the G gene of RSV were extracted from the gel, subcloned into pGEM-t-easy vector, and submitted for sequencing (GenoTech, Daejeon, Korea). Raw sequence data were assembled using Contig Express and G ORF sequences were aligned with the two reference sequences, HRSV A2 (AF035006.1) for HRSV-A and B1 (AF013254) for HRSV-B, by using the AlignX software (Invitrogen). The phylogenetic tree was constructed by the neighboring-joining method with Kimura two-parameter distances by using MEGA version 5. The reliability of internal branches was assessed by 1000 bootstrap replications and the *p*-distance substitution model.

### Preparation of HRSV stock

Large-scale RSV isolate was prepared by infecting thirty 150-mm dishes of monolayered HEp-2 cells (ATCC, Manassas, VA) with the previously prepared small-scale isolate stock at MOI of 0.01. Virus was harvested when the cytopathic effect was over 60% (usually 4 days after inoculation) and then titrated for infectivity by plaque assay.

### Mice immunization and virus challenge

Four- to six-week-old, specific-pathogen free, female BALB/c mice were purchased from Charles River Laboratories (Orient Bio, Korea). Construction and preparation of rAd vaccines have been described elsewhere [14]. For intranasal immunization, mice were inoculated with 5×10^6^ PFU of replication-defective rAd/3xG vaccine or control vaccine in a volume of 70 µl to the left nostril. Three to four weeks later, the immunized mice were challenged i.n. with 1×10^6^ PFU of KR/A/09-8 or 2×10^6^ PFU of KR/B/10-12 isolate.

### ELISA

Heparinized blood was obtained by retro-orbital eye bleeding. The collected blood was centrifuged, and serum was obtained and stored at −20°C. Broncho-alveolar lavage (BAL) fluid was obtained by washing the lung airway three times with 0.8 ml of 0.85% saline/0.6 mM EDTA. Specific antibody titers for RSV from immunized mice were measured by direct ELISA. Briefly, 96-well plates were coated with 1×10^3^ PFU of purified RSV A2 virus diluted in 100 µl of PBS overnight, and blocked with PBS containing 1% non-fat milk and 0.05% Tween-20 for 2 h. Sera or lavage fluids were then added in serial dilutions and incubated for 2 h. The plates were washed with PBS containing 0.05% Tween-20 five times and incubated for 1 h with various dilutions of HRP-conjugated affinity-purified rabbit anti-mouse total IgG or anti-mouse IgA secondary antibodies (Zymed Laboratories, San Francisco, CA). The plates were washed three times, developed with 3,3′,5,5′-tetramethylbenzidine, stopped with 1 M H_3_PO_4_, and analyzed at 450 nm by a Thermo ELISA plate reader.

### RSV titer in the lung

Four days after RSV challenge, individual mice were euthanized and the lung tissues were removed into minimum essential medium. The tissues were then processed through a 70-µm cell strainer (BD Labware, Franklin Lakes, NJ) with 2.5 ml of minimum essential media. The supernatants were collected and RSV titers in the supernatants were measured by plaque assay on 90% confluent HEp-2 cell monolayer. The data are expressed as the PFU per gram of lung tissue. The limit of detection was 200 PFU/g of lungs.

### Data analysis

The data were compared in an unpaired, two-tailed Student's t-test. The difference was considered statistically significant when the P value was ≤0.05.

### Ethics statement

Written informed consent was obtained from the parents of pediatric patients and the study was approved by the Institutional Review Boards of the Ewha Womans University Mokdong Hospital. All animal experiments were also approved by Ewha Womans University's Institutional Animal Care and Use Committee (Approval ID: 2010-9-4).

## Results

### Detection of HRSV in nasopharyngeal samples

**Figure 1:**
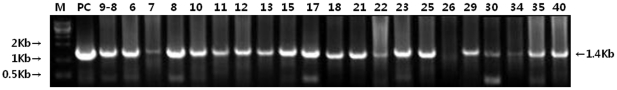
RT-PCR analysis of infected HEp-2 cell lysates. Agarose gel electrophoresis of RT-PCR products was performed to detect G gene. cDNA from cell lysates of RSV A2-infected HEp-2 cells was used as a positive control. Isolate numbers are indicated for each lane. M: size marker, PC: positive control.

From November 2008 to April 2010, 70 nasopharyngeal aspirates were obtained from children who were admitted to Ewha Womans University Mokdong Hospital with symptoms of acute LRTI. To detect HRSV in clinical samples, we performed RT-PCR assays with RSV-specific primer sets. The primers were designed to amplify three viral genes: NS2, SH and G. As previously described, NS2 is the relatively conserved viral gene whereas SH and G genes vary between subgroups of RSV. In order to amplify SH or G gene regardless of subtype, specific primers were designed based on the relatively conserved flanking regions of the SH or G gene. The primer sequences are listed in the Materials and Methods. Using these primer sets, we successfully detected and amplified each gene regardless of subtype. Of the 70 nasal samples, 21 samples (30%) were positive for HRSV G (Fig. 1).

### Phylogenetic and nucleotide matching analyses based on G gene sequences

**Figure 2:**
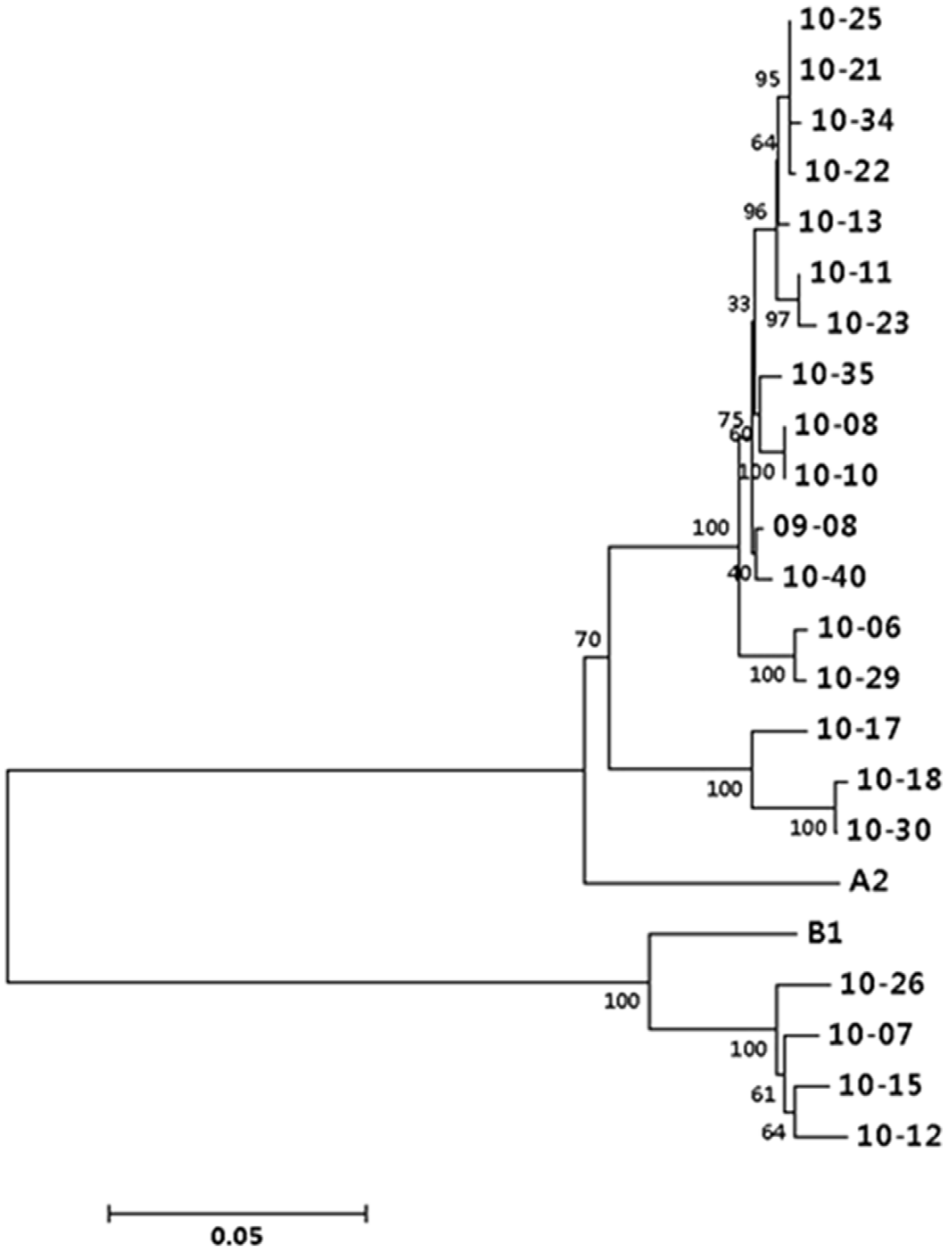
Phylogenetic analysis of the G gene of HRSV isolates. A phylogenetic tree was built by the neighbor-joining method in MEGA version 5 based on the sequence of the whole G ORF from HRSV isolates. Reference sequences for each subtype, A2 and B1, were also included. All HRSV isolates were labeled by the year of isolation and the order of sampling. The numbers at the branch nodes indicate the bootstrap value. Bootstrap values were calculated with 1000 replicates. The scale bar represents the percentage of nucleotide changes.

RSV G-positive samples were analyzed to examine the phylogenetic relationship based on the sequence of the G gene. Phylogenetic analyses showed that 17 of the 21 isolates (09-8, 10-6, 10-7, 10-8, 10-10, 10-11, 10-12, 10-13, 10-15, 10-17, 10-18, 10-21, 10-22, 10-23, 10-25, 10-26, 10-29, 10-30, 10-34, 10-35 and 10-40) belonged to the HRSV-A subgroup and 4 (10-7, 10-12, 10-15, 10-26) belonged to the HRSV-B subgroup. This result showed that two subgroups of RSV co-circulated in the same epidemic period and that HRSV-A was more prevalent than HRSV-B in the 2009/2010 season. Furthermore, the HRSV-A isolates could be broadly divided into 3 clusters with sufficiently high bootstrap values (Fig. 2).

Through the phylogenetic analysis, we also examined the sequence differences between the isolates and two prototype strains, RSV A2 and B1, by pair-wise comparison of the whole sequence of the G ORF. The sequence matching data showed that nucleotide sequence homology was 89∼91% between RSV A2 and HRSV-A isolates, while the homology was 92∼93% between RSV B1 and HRSV-B isolates.

**Figure 3:**
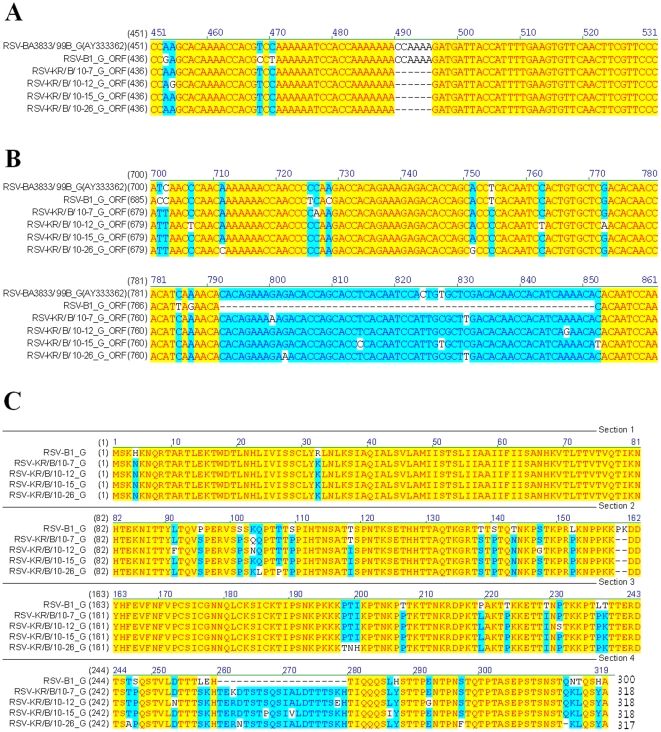
Characteristic features of G sequences of HRSV-B isolates. The G ORF sequences of four HRSV-B isolates were compared with those of HRSV B1 and genotype BA by pairwise comparison. Dashes indicate the deletion of 6 nucleotides in HRSV-B isolates (A) and the absence of a 60-nucleotide insertion in HRSV B1 reference strain (B). Numbers represent the registered nucleotide sequence of RSV-BA3833/99B_G (AY333362) from the PubMed database. Deduced amino acid sequences of the G gene are also aligned with that of HRSV B1 (C). Each predicted G protein length is also indicated at the end of the sequences.

Intriguingly, drastic changes such as deletions and insertions were observed in HRSV-B isolates. All of the HRSV-B isolates identified in this study showed consecutive deletion of 6 bases between nucleotides 491 and 496 and consecutive insertion of 60 bases between nucleotides 792 and 851, when compared to the reference RSV B1 strain (Fig. 3A and B, respectively). Similar 60-nucleotide duplication was also observed in BA-genotype isolates of Buenos Aires, Japan, and Kenya [15], [16], [17]. However, unique deletion of 6 bases between nucleotides 491 to 496 has not been reported in the BA genotype. Furthermore, these significant genetic changes suggest that some antigenic changes escaping immune pressure may have occurred following these deletions and duplications of sequences found in Korean subgroup B isolates (Fig. 3C).

The previous study reported that a *PstI* restriction enzyme site was uniquely conserved in the G gene of most Korean HRSV-A isolates, whereas none of the HRSV-B isolates had a *PstI* site [18]. Consistent with this report, all of the HRSV-A isolates in this study contained this conserved *PstI* site (data not shown).

### Adaptation and large-scale amplification of HRSV isolates in HEp-2 cells

**Figure 4:**
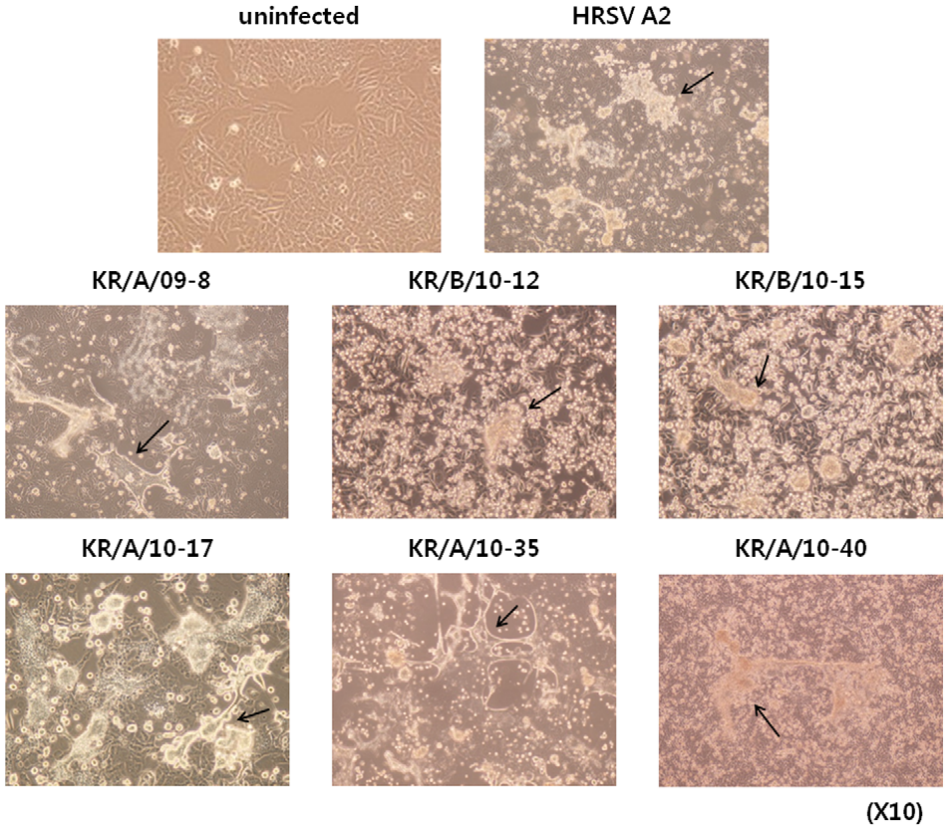
Morphological characteristics of HEp-2 cells infected by HRSV isolates. Cytopathic effects of HRSV isolates were observed 3∼5 days after infecting HEp-2 cells. Syncytia (indicated by arrows) were more apparent and larger in cultures infected with HRSV-A isolates than HRSV-B isolates. Magnification, ×10.

**Figure 5:**
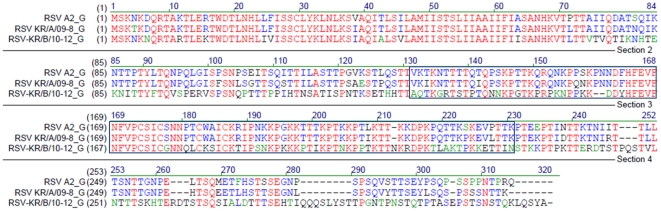
Homology comparison of HRSV A2, KR/A/09-8, and KR/B/10-12 G proteins at deduced amino acid level. The deduced amino acid sequences of G gene of KR/A/09-8 and KR/B/10-12 are compared with that of HRSV A2 by sequence alignment. 87% of amino acid sequence of KR/A/09-8 G and 53% of KR/B/10-12 G are matched with that of HRSV A2 G. The boxed region indicates the central core domain of G employed in the rAd/3xG vaccine. Identical residues are indicated by red and conservative or weakly similar changes are indicated by blue or green, respectively.

To stably adapt patient-isolated viruses to the HEp-2 cell line, viruses were subcultured for more than 10 passages. Several HRSV isolates infecting HEp-2 cells showed distinct cytopathic effects (CPE), characterized by formation of syncytia. Of the 25 RSV-positive samples, we selected 6 isolates (KR/A/09-8, KR/B/10-12, KR/B/10-15, KR/A/10-17, KR/A/10-35, KR/A/10-40) for adaptation and amplification in cell culture because these viral samples showed more extensive CPE than other samples, making it easier to examine the morphological changes after infection (Fig. 4). Interestingly, morphological changes induced by the isolates were somewhat different between subgroups. For example, cells infected with HRSV-A isolates produced relatively larger and more syncytia than HRSV-B isolates. To exclude the possibility that cultures were cross-contaminated with other viruses, each virus was isolated and amplified from plaques of early passage, and confirmed by immunohistochemistry with RSV-specific antibody (Figure S1). After adaptation, one HRSV-A isolate, KR/A/09-8, and one HRSV-B isolate, KR/B/10-12, were selected for large-scale amplification since these two isolates showed faster growth kinetics, higher titer, and more distinct CPE appearance than other isolates. KR/A/09-8 and KR/B/10-12 have ∼87% and ∼53% deduced amino acid identity compared with the G sequence of the reference HRSV A2 strain, respectively (Fig. 5).

### Protective efficacy of RSV vaccine against challenge of HRSV isolates

**Figure 6:**
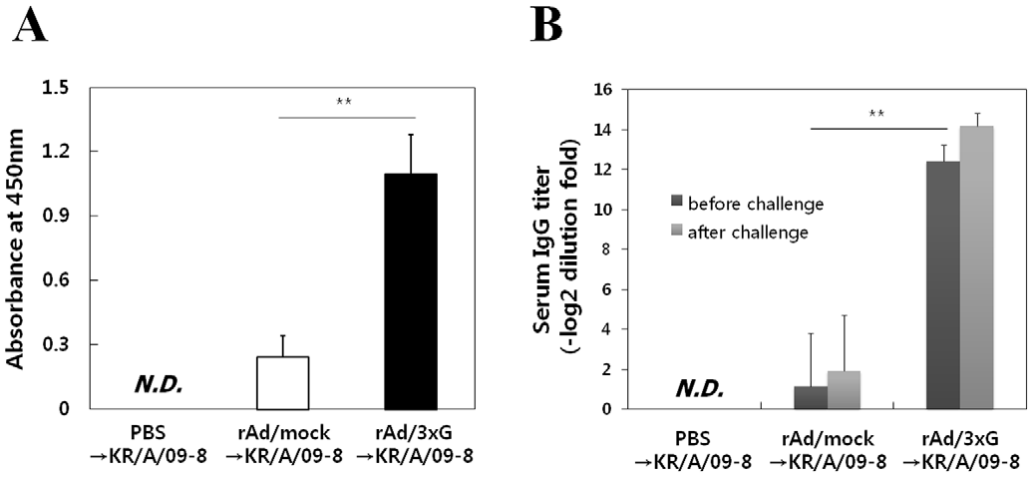
Characterization of humoral responses before and after KR/A/09-8 challenge. Balb/c mice were i.n. immunized with 5×10^6^ PFU of rAd/3xG or rAd/mock. Immune mice were challenged with KR/A/09-8 at 3 weeks after immunization. Average IgA titers were measured in the BAL fluid 4 days after challenge (A). Systemic anti-RSV IgG antibody titers were measured by serum ELISA 3 weeks after immunization (before challenge) or 4 days after challenge (B). The results represent Log2 endpoint values from five individual mice. The results are representative of three independent experiments. N.D., not detected. *, *p*<0.01; **, *p*<0.001.

Previously, our laboratory reported that single mucosal immunization of the recombinant adenovirus-based RSV vaccine, rAd/3xG, elicited protective immunity against RSV [14]. This study showed that a single intranasal immunization of rAd/3xG induced strong serum IgG response, mucosal IgA response, and long-term protection following RSV A2 virus challenge. To examine the protective efficacy of rAd/3xG against the HRSV isolates, BALB/c mice were immunized once via the intranasal route with 5×10^6^ PFU of rAd/3xG or rAd/mock as a control. These mice were then challenged with 1×10^6^ PFU of KR/A/09-8 or 2×10^6^ PFU of KR/B/10-12 at 3 weeks after immunization. Four days after challenge with KR/A/09-8, the levels of mucosal IgA in BAL fluid and serum IgG of rAd/3xG-immune mice were much significantly higher than in the PBS control or rAd/mock-immunized group (Fig. 6A and 6B).

**Figure 7:**
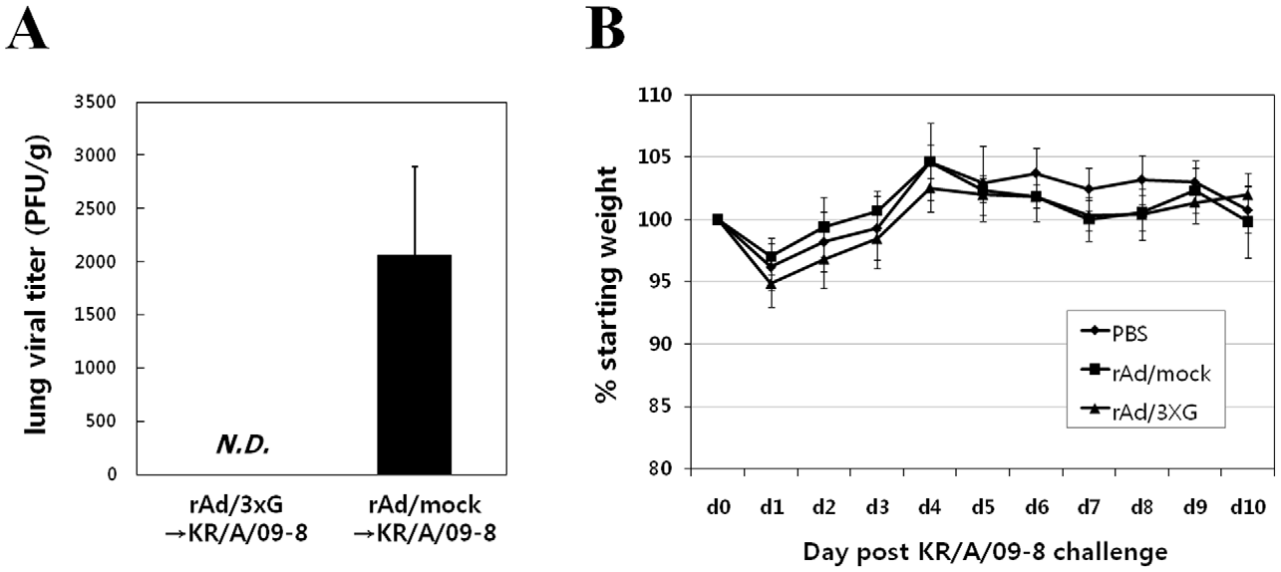
Immune protection from KR/A/09-8 challenge by vaccination with rAd/3xG. Each group of immunized mice was challenged with 1×10^6^ PFU of KR/A/09-8 at 3 weeks after immunization. The levels of viral replication in the lungs were determined by plaque assay on day 4. The results are expressed as the mean ± SEM from five mice for each group. The limit of detection is 200 PFU/g of lungs. N.D., not detected. The results are representative of two independent experiments. The same groups of immune mice were challenged with KR/A/09-8 and then weighed each day (B). Results are expressed as the mean ± SEM from 5 mice for each group.

**Figure 8:**
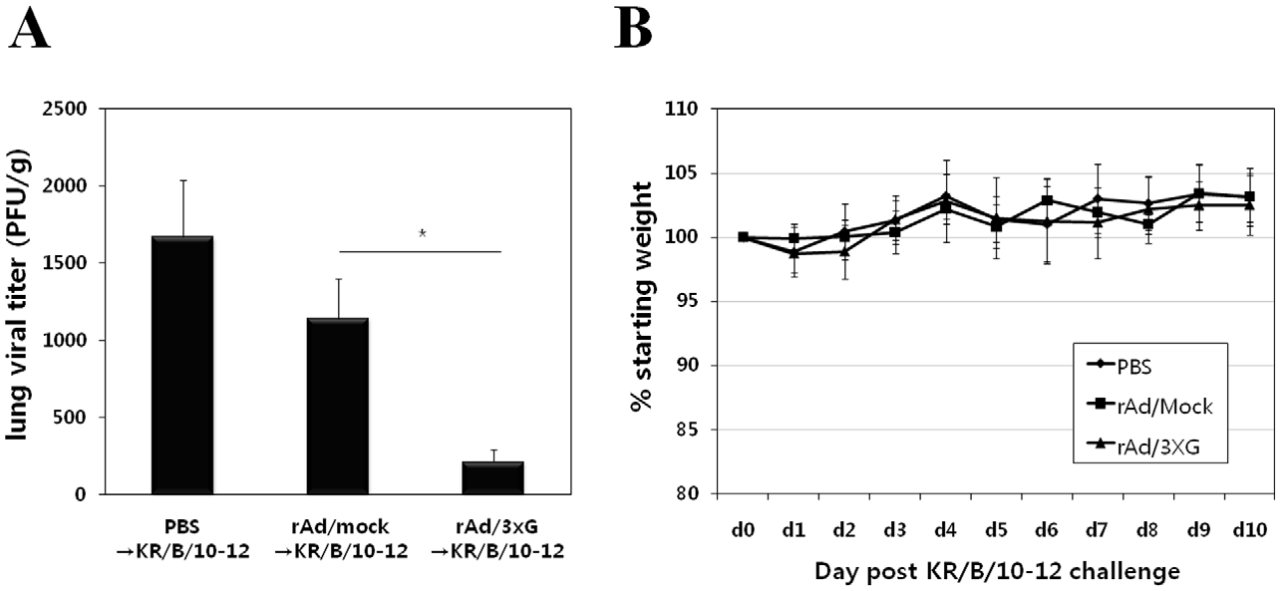
Immune protection from KR/B/10-12 challenge by vaccination with rAd/3xG. Each group of immunized mice was challenged with 2×10^6^ PFU of KR/B/10-12 at 3 weeks after immunization. The levels of viral replication in the lungs were determined by plaque assay on day 4. The results are expressed as the mean ± SEM from five mice for each group. The limit of detection is 200 PFU/g of lungs. The results are representative of two independent experiments. The same groups of immune mice were challenged with KR/B/10-12 and then weighed each day (B). Results are expressed as the mean ± SEM from 5 mice for each group. *, *p*<0.01.

To test the protective efficacy of rAd/3xG immunization against KR/A/09-8 challenge, lung homogenates were prepared on day 4 after challenge, at the peak of viral replication, and the viral plaque assay was performed with lung homogenates. There was no detectable virus in the homogenates of rAd/3xG-immune mice, indicating that single intranasal immunization of rAd/3xG confers perfect protection against KR/A/09-8 infection (Fig. 7A). There was no significant weight loss upon KR/A/09-8 challenge in rAd/3xG-immune or control mice (Fig. 7B). These results demonstrate that RSV A2-based rAd/3xG vaccine induces broad protective immunity that is effective against a HRSV-A isolate with ∼89% homology in the G protein. Vaccination of rAd/3xG also provided potent protection against KR/B/10-12 B type isolate challenge (Fig. 8A), even though the level of protection was not perfect as in KR/A/09-8 challenge. There was neither significant weight loss upon KR/B/10-12 challenge in rAd/3xG-immune or control mice (Fig. 8B). These results demonstrate that RSV A2-based rAd/3xG vaccine induces cross-protective immunity that is also effective against a HRSV-B isolate with ∼53% homology in the G protein.

## Discussion

HRSV is the most common viral pathogen in infants and young children with acute LRTI, and repeated infection often occurs throughout life. Despite its importance, there have been limited studies about the prevalence and characterization of HRSV in Korea. In the present study, we have detected HRSV in samples obtained from young children less than 3 years of age. The nasal samples were collected from children with acute LRTI during the period from November 2008 to April 2010. The G sequences of HRSV isolates were prepared by RT-PCR with the G gene-specific primer set. In many studies, primers used to amplify the G sequence were from the 3′ distal region of the G gene, which is known as the second variable region, and different primers had to be used for each strain of RSV. We also designed primers that could amplify the whole G gene regardless of strain so as to examine the genetic variability of the G gene on the whole using one primer pair. Actually, the primer set for the G gene was more efficacious in detecting HRSV by RT-PCR than the other primer sets (Fig. 1 and data not shown). However, we cannot completely rule out the possibility that the primers used might have missed some circulating genetic variants of HRSV in Korea, even though the primers were designed on the most conserved regions between the two subgroups.

Several viral samples exhibited similar cytopathic effects in cell culture, but HRSV was not detected in the RT-PCR assay. This discordance in the viral culture and RT-PCR results may be due to another respiratory virus infection, such as human metapneumovirus (HMPV), parainfluenza virus, or rhinovirus. Previous studies have indicated that the epidemics of respiratory viral infection largely overlap, and co-infection of several respiratory viruses frequently occurs. In particular, it is reported that dual infection of HMPV and HRSV occurs at a relatively high frequency (up to 70%) [19].

Our genetic analysis data demonstrated that two subgroups of HRSV co-circulate in the same period, and subgroup A was more prevalent than subgroup B (81% vs. 19%, respectively). Nucleotide sequence comparisons among each group of isolates showed that genetic variability was higher among the subgroup A isolates (up to 10%) than among the subgroup B isolates (∼3%). This result is in accordance with previous reports that more extensive variability of the G gene was observed in subgroup A [20], [21], [22]. Moreover, the higher genetic variability among subgroup A isolates could explain the predominance of subgroup A worldwide [23], [24].

G protein is one of the targets of neutralizing antibodies, and its capacity to accommodate drastic antigenic changes has been well illustrated by the presence of escape mutants [25], [26], [27], [28]. As shown in Fig. 3B, our data demonstrate that the G gene of all subgroup B isolates has 60-nucleotide duplication at the same position (corresponding to nucleotide 792 and 851 of the reference RSV B1 strain). Although there were other minor alterations at the nucleotide level, the inserted 60 nucleotides were almost identical among the 4 subgroup B isolates. Previously, Trento *et al.* first reported novel isolates from Buenos Aires, Argentina, belonging to a new genotype named BA, which has 60-nucleotide duplication at the position of nucleotide 792 [16]. In addition, Nagai *et al.* reported that four RSV isolates that belong to subgroup B had 60 extra nucleotides that were nearly identical in sequence and position of insertion with those of the isolates found in Argentina [15]. The prevalence of the new BA genotype virus suggests that the duplication provides a selective advantage over other HSRV-B lacking duplication. It has yet to be determined whether and how this 60-nucleotide duplication gives any selective advantage and/or boosts virus fitness during virus evolution.

The RSV G glycoprotein is thought to be a good candidate antigen, since it is the target of neutralizing antibodies and is under immune pressure [29]. Various strategies have been employed to develop a safe and effective RSV vaccine. Among these, vaccine candidates using live viral vectors such as replicating vaccinia virus and non-replicating adenovirus have been shown to be immunogenic and protective to varying degrees in animal models [30]. Our study shows that the RSV vaccine, rAd/3xG, which is constructed based on the G sequence of RSV A2, has cross-protective efficacy against field-isolated KR/A/09-8 and KR/B/10-12 in a murine model. The core region of the G sequence employed in rAd/3xG vaccine (amino acids 131–230) is relatively well-conserved, and there are only 7% differences in the amino acid level between the region of RSV A2 G and KR/A/09-8 G. However, there are ∼47% differences between the sequence of A2 and KR/B/10-12, which resulted in partial protection against KR/B/10-12 challenge. Thus, it is likely that similarity of the antigenic determinants within this conserved G core region correlates with the effectiveness of cross-protective immunity induced by rAd/3xG vaccination, which confers more effective protection against HRSV-A infection than HRSV-B infection. Our findings clearly emphasize that the sequence identity between vaccines and infecting RSV is important for the development of more “universal” RSV vaccines. It will be interesting to investigate how immunity induced by rAd/3xG expressing subgroup B sequence is effective against HRSV-B infection. Our data suggest that multiple G components representing subgroup A and B may be required for development of broadly protective HRSV vaccines.

## Supporting Information

Figure S1 **Verification of HRSV infection by immunohistochemistry.** HEp-2 cells infected with RSV A2 or the indicated isolates were fixed, blocked with FBS, and stained with goat anti-HRSV antibody conjugated to HRP (US Biological). Spots were developed with 3-amino-9-ethylcarbazole substrate.
(TIF)
