# Supplementary figures and images for: Evaluation of Protective Efficacy of Respiratory Syncytial Virus Vaccine against A and B Subgroup Human Isolates in Korea
Source: PLoS One. 2011 Sep 7;6(9):e23797. doi: 10.1371/journal.pone.0023797 (PMC3168431; doi:10.1371/journal.pone.0023797)

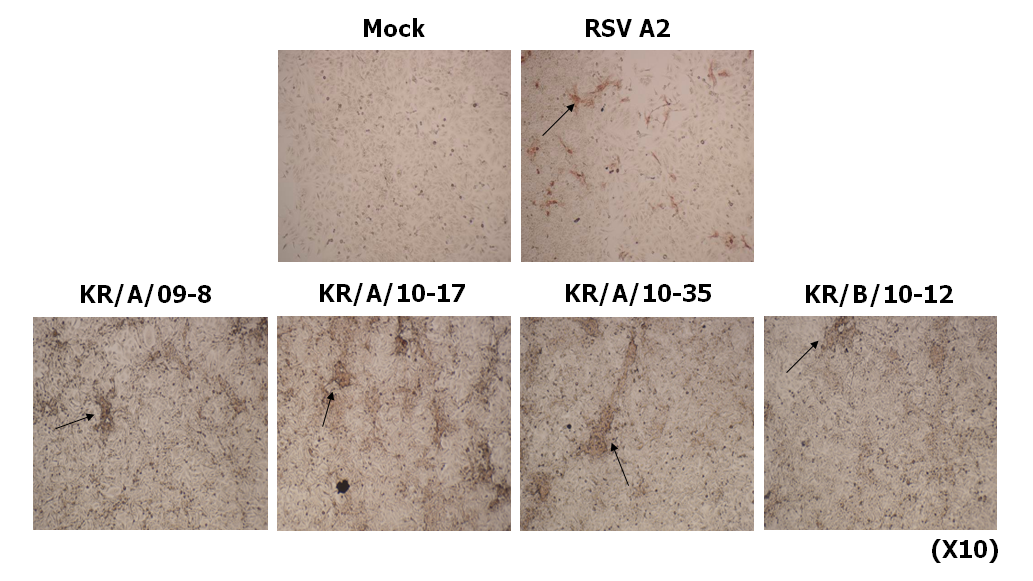

Supplement: Figure S1 — Verification of HRSV infection by immunohistochemistry. HEp-2 cells infected with RSV A2 or the indicated isolates were fixed, blocked with FBS, and stained with goat anti-HRSV antibody conjugated to HRP (US Biological). Spots were developed with 3-amino-9-ethylcarbazole substrate. (TIF) [file pone.0023797.s001.tif]
